# Initial Experience of Clinical Use of [^99m^Tc]Tc-PSMA-T4 in Patients with Prostate Cancer. A Pilot Study

**DOI:** 10.3390/ph14111107

**Published:** 2021-10-29

**Authors:** Jarosław B. Ćwikła, Marek Roslan, Iwona Skoneczna, Monika Kempińska-Wróbel, Michał Maurin, Wojciech Rogowski, Barbara Janota, Anna Szarowicz, Piotr Garnuszek

**Affiliations:** 1 Department of Cardiology and Internal Medicine, School of Medicine, University of Warmia and Mazury, 11-041 Olsztyn, Poland; m.k.w.bioscience@gmail.com; 2 Diagnostic and Therapy Center—Gammed, 02-351 Warsaw, Poland; 3 Department of Urology, School of Medicine, University of Warmia and Mazury, 11-041 Olsztyn, Poland; marek.roslan@uwm.edu.pl; 4 Department of Urology, Grochowski Hospital, 04-073 Warsaw, Poland; iaskoneczna@gmail.com; 5 Department of Urology, Maria Sklodowska Curie National Research Institute of Oncology, 02-781 Warsaw, Poland; 6 National Centre for Nuclear Research, Radioisotope Centre POLATOM, 05-400 Otwock, Poland; michal.maurin@polatom.pl (M.M.); barbara.janota@polatom.pl (B.J.); piotr.garnuszek@polatom.pl (P.G.); 7 Department of Urology, Hospital Ministry of Internal Affairs, 02-507 Warsaw, Poland; wojciech.rogowski@gmail.com; 8 GE Healthcare, 02-583 Warsaw, Poland; anna.szarowicz@ge.com

**Keywords:** prostate cancer, molecular imaging, prostate specific membrane antigen (PSMA), [^99m^Tc]Tc-PSMA-T4

## Abstract

Numerous different molecules of prostate-specific membrane antigen (PSMA) ligands are used to detect prostate cancer (PCa); most approaches utilize gallium PET and a few reports describe the role of SPECT/CT. [^99m^Tc]Tc-PSMA-T4 is a new radiopharmaceutical designed for the diagnosis of patients with PCa. We conducted a single site, prospective, preliminary case series study that included 31 patients with PCa; all had undergone clinical, biochemical or imaging examination and exhibited clear or suspicious active disease or clinical/biochemical recurrence of PCa. Whole-body (WB) SPECT/CT after i.v. administration of [^99m^Tc]Tc-PSMA-T4 was utilized; acquisition images were obtained at three time points. The clinical value of the images was assessed in regard to the evaluation of tumor extent in patients with confirmed PC that qualified for initial therapy and the evaluation of tumor recurrence; both provided encouraging results. The late acquisition of WB-SPECT resulted in better lesions delineation. The results of the analysis of the sensitivity/specificity were: 92%/100% in cases of primary cancer, 83%/100% in terms of pelvic lymph nodes disease, 100%/95% in other lymph nodes and soft tissue involvement, respectively, and bone mets were both 100%. An oncotropic SPECT [^99m^Tc]Tc-PSMA-T4 can help in selecting a rational therapeutic strategy for a patient with an initial diagnosis of PCa by assessing the extent of cancer and also after complex radical or palliative therapy in case of biochemical recurrence for re-staging.

## 1. Introduction

Optimal clinical decision-making for men with prostate cancer (PCa) requires the analysis of many factors, including prostate-specific antigen (PSA) assessment and performing different kinds of scans and other molecular or genetic testing. We are not able to distinguish between low and high-risk patients with PCa on the basis of PSA results only, so this biomarker cannot be used to select the most appropriate treatment for patients [1]. 

Current standard diagnostic imaging, including structural imaging (CT or MRI) and standard bone scan are not optimal for stage evaluation, in detecting aspects of local disease, lymphatic spread or assessment of distant metastatic disease. Pelvic MRI with PI-RADS v.2 evaluation of loco-regional extent is has now become the imaging standard before “Tru-Cut” biopsy, but this is not informative in regard to the extra pelvic extent of disease. The usually recommended contrast-enhanced CT of chest, abdomen and pelvis is of low sensitivity in the assessment of local spread, including the presence of recurrence in the primary location and involvement or recurrence in lymph nodes [2,3].

The bone scan is established as the standard procedure for the confirmation or exclusion of bone metastatic disease. However, the relatively high sensitivity of bone scans (greater than 90%) is combined with a specificity of less than 50%; additionally, bone scan lacks information about soft tissue involvement, particularly the presence of involved lymph nodes and metastases to other soft tissues. Additionally, bone scans indicate bone metabolic exchange rather than the presence of cancer cells within bone marrow, so low volume disease or lesions with minimal osteoblastic activity may not be visible on the standard bone scan [4].

Using standard imaging with bone scans and CT, even together, leads to imprecise cancer detection and limits the selection of optimal strategies for the patient’s management, which is of importance in cases of limited and extended disease. With new metastasis-directed approaches and new hormonal agents, the early detection of systemic disease with better imaging is crucial for further therapy selection. In particular, patients with high-risk PCa may be diagnosed earlier, with limited local or distal spread (oligometastatic disease) and may be good candidates for a more radical treatment approach [5,6,7]. 

Currently, there are several new molecular imaging approaches that give more accurate information about the stage of disease and help to select patients into appropriate therapy. Most of them utilize PET technology and use ^68^Ga or ^18^F radioisotopes, which are conjugated with prostate-specific membrane antigen (PSMA) ligands [8,9,10,11,12]. The PSMA known as glutamate carboxypeptidase II, is a transmembrane, 750 amino acid, type II glycoprotein, which is expressed by almost all primary PCa and metastatic diseases as well [8]. PSMA is highly homologous to N-acetylated R-linked acidic dipeptidase, a neuropeptidase that produces the neurotransmitter glutamate and N-acetylaspartate through the hydrolysis of N-acetylaspartylglutamate [13]. 

The expression of PSMA is further increased in poorly differentiated, metastatic lesions, at hormone-sensitive and castration-resistant stages and may serve as an early progression indicator [14,15,16]. In addition, the increased expression of PSMA in primary PCa correlates with other adverse traditional prognostic factors and independently predicts worse disease outcome [17]. Therefore, PSMA seems to be an ideal target for developing imaging biomarkers for PCa [18,19,20,21]. 

Investigators at Molecular Insight Pharmaceuticals (MIP) developed a series of novel glutamateurea (Glu-urea) amino acid heterodimeric inhibitors of PSMA for developing SPECT radiopharmaceuticals using ^123^I or ^99m^Tc [22,23,24]. In recent years, many PSMA inhibitor analogues containing various chelating systems for technetium-99m coordination have been synthesized [22,23,24,25,26,27,28,29,30,31]. ^99m^Tc is the optimal radionuclide for developing SPECT radiopharmaceuticals. Additionally, using SPECT technologies means this approach could be used in any nuclear medicine department, and also in those without PET. SPECT technology could also be a very useful approach in radio-guided surgery with a standard gamma probe to detect local and regional lymph nodes involvement [32]. Due to the high incidence of PCa, SPECT technology could be used upfront to select patients for appropriate therapy and currently, there is also an emerging interest in PSMA radio-guided surgery to improve the efficacy of radical surgery. Investigators from the National Center for Nuclear Research Radioisotopes Centre POLATOM developed a new radioligand, PSMA-T4 (Glu-CO-Lys-L-Trp-4-Amc-HYNIC) (C_37_H_49_N_9_O_10_ with a molecular weight (MW) of 779.36 g/mol), which potentially can be used in the diagnosis of PCa patients [33]. The first clinical use of this radiopharmaceutical in humans was presented by Sergieva et al. [34]; the same team also reported its clinical use in patients with recurrent PCa [35]. 

The goal of this study was to report our initial experience of the clinical use of [^99m^Tc]Tc-labeled PSMA-T4 using WB-SPECT/CT technology with patients with confirmed PCa.

## 2. Results

### 2.1. Subject Population

Overall, 31 subjects were included in the study, all had pathological confirmation of PCa, with a Gleason score from 6 to 10. The mean age was 68.1 years old (range: 53–87). Clinical, pathological and biochemical details of the study group are presented in Table A1 and Table A2. The mean administered activity of [^99m^Tc]Tc-PSMA-T4 (MBq) was 435.8 MBq (range 310–530 MBq, SD +/− 49.2 MBq). After administration there was no clinically detectable pharmacologic effects, that is, adverse events (AEs).

In the study group, eight subjects (26%) were imaged for an evaluation of already present active disease to measure the current extent and metabolic activity (palliative settings), three patients (10%) for an evaluation of disease extent before radical therapy, while the rest of the patients were imaged for evaluation of suspected recurrent disease.

### 2.2. Biodistribution of Radiopharmaceutical

Normal biodistribution of the radiopharmaceutical with a high activity background was observed in the liver, spleen, kidneys, salivary and lacrimal glands. Low diffuse activity was seen in the bowel, particularly the left colon; additionally, low diffuse activity, particularly in delayed images, was seen in the urinary bladder. After intravenous administration, [^99m^Tc]Tc-PSMA-T4 is rapidly eliminated from the blood. After 10 min, accumulation of [^99m^Tc]Tc-PSMA-T4 can be seen in the main organs, i.e., liver, spleen and kidneys as well as in tumor lesions expressing PSMA [33]. The radiopharmaceutical is excreted mainly by the renal route with a small contribution by hepatic excretion. [^99m^Tc]Tc-PSMA-T4 is rapidly eliminated from the blood, so the images taken 1 h after injection of the radiotracer are of good quality. The activity accumulated in the blood cells is below 5% regardless of time after injection [33,34,35]. The images obtained after 3–4 h and 5–7 h are very similar in terms of clinical utility.

### 2.3. Imaging Studies 

**Figure 1:**
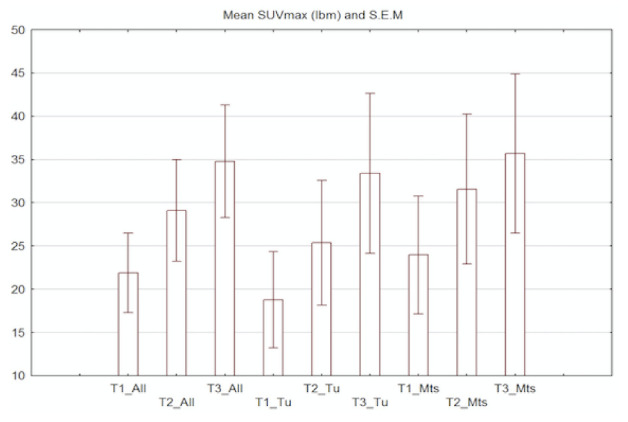
The summarized results of the mean value of SUVmax (lbm) in data sets from three acquisitions (T1—early 1.0–1.5h, T2—3.0–4.0 h and late T3—6.0–7.0 h) in selected patients with active disease including the mean value of SUVmax (lbm) for all detected and selected lesions (T1–T3_All), in those patients with the presence of prostate tumors (T1–T3_Tu) and in those subjects with metastatic disease including lymph nodes involvement and bone metastasis (T1–T3_Mts).

We performed 3 acquisition scans of WB-SPECT/CT in our first 20 patients, as per the protocol, and very good accumulation in all three timeframe scans was noted, but in the delayed third images, we found a higher accumulation of radiotracer based on SUVmax (lbm) analysis. The results indicated that delayed images of [^99m^Tc]Tc-PSMA-T4 scans have higher SUVmax in all analyzed VOIs with pathological uptake of radiopharmaceutical, independent of localization in both primary tumors in the prostate and secondaries. The higher accumulation was seen in all lesions (*n* = 25 lesions) in the third acquisition compared to the first and second acquisitions (*p* < 0.001, Wilcoxon paired match test) and also in prostate glands (*n* = 10 cancers) (*p* < 0.05). The difference in SUVmax (lbm) was also seen in third acquisitions compared to the first and second in involved lymph nodes and bone mets. The graphical summarized results of the mean value of SUVmax (lbm) in the data sets of three acquisitions (T1, T2 and T3) in selected patients, that is, those with active disease including the mean value of SUVmax (lbm) are presented in Figure 1.

**Figure 2:**
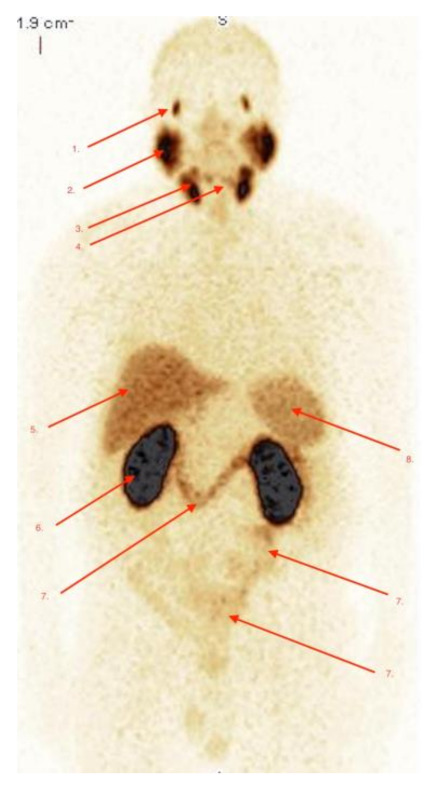
Physiological biodistribution of [^99m^Tc]Tc-PSMA-T4 in patient without active pathology due to PCa. The physiological uptake of radio-tracer is seen in the coronal view of the WB-SPECT-scan (MIP-Multi-Image-Projections), 5h after i.v. injection of 459MBq of [^99m^Tc]Tc-PSMA-T4. The very high uptake of radiotracer is seen in lacrimal glands (1), parotid and submandibular glands (2) and (3), and in the present case, also in additional submandibular glands (4), and additional high physiological uptake of radio-tracer is seen in both kidneys (6). Less intensive physiological uptake of radiopharmaceutical is also seen in the liver (5), large bowel (7) and spleen (8).

Physiological distribution of radiotracer was observed in the same location as in patients scanned with [^68^Ga]Ga-PSMA-11. An example of physiological accumulation of ^99m^Tc]Tc-PSMA-T4 is shown in Figure 2, and is consistent with physiological accumulation [^68^Ga]Ga-PSMA-11. The very high uptake of [^99m^Tc]Tc-PSMA-T4 is seen in the lacrimal gland (1), parotid and submandibular glands (2) and (3), and in the presented case, also in additional submandibular glands (4), with additional high physiological uptake of radiotracer seen in both kidneys (6). Less intensive physiological uptake of radiotracer is also seen in the liver (5), large bowel (7) and spleen (8).

### 2.4. Detection of Active Pathology in Patients

**Figure 3:**
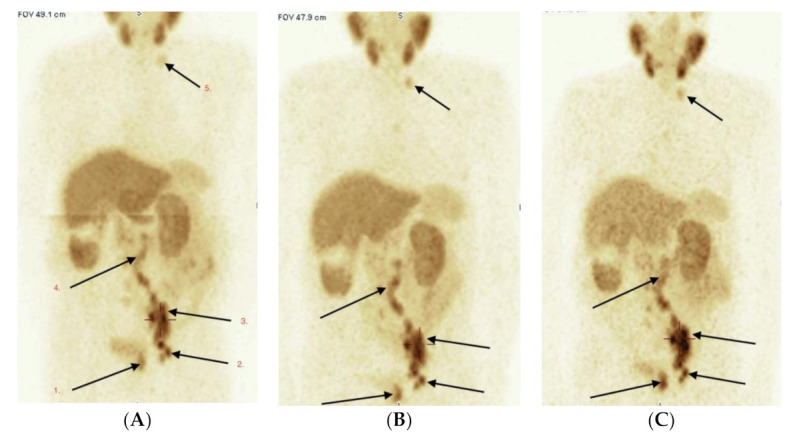
(**A**–**C**) Male 87 year, adenoca G3; GlS = 8 (4 + 4). PSA 228.0 ng/mL. Evaluation of tumor extent. Three scans of WB-SPECT/CT acquisition after 1 h (**A**), 3 h (**B**) and 6 h (**C**) after radio-tracer i.v. administration. Pathological high uptake of [99mTc]Tc-PSMA-T4 is seen in primary PCa—left lobe of prostate (1), multiple lymph nodes: left obturator (2), left external and common iliac (3), paraaortic left and right (4) and the left neck (5). No bone lesions were detected in current PSMA, bone and CT scans.

There were 13 patients with a definitive active pathology with high accumulation of radiotracer on a semiquantitative scale of grade 4 and 5. There was an additional 8 patients with suspicious radiopharmaceutical accumulation with the probability of PCa lesions. In the first group, all had confirmation of active pathology as PCa, in the second group. 6 of 8 had pathological confirmation of PCa. Overall, 19 subjects (61%) had a positive scan in terms of active pathology, which was verified by pathology and during clinical or biochemical follow-up for at least 6 months (Figure 3A–C).

We obtained equivocal results for two patients, in one case we revealed non-small lung carcinoma, which was confirmed later by pathology, so our result was false positive due to active lung carcinoma. Due to extended lung disease, further therapy for the PCa was abandoned. The second patient had a nodal disease and remained in active clinical and biochemical follow-up.

In the group with positive scans, 10 patients had very high, avid radiotracer accumulation (grade 5), while the other 3 had significant (grade 4) uptake. In 15 patients with the presence of prostate gland, 11 had true positive results (TP) with the presence of focal pathological uptake within the prostate. Three results were true negative in 3 patients after radical radiotherapy with no active pathology within the prostate seen on imaging and during the follow-up. A single subject had a false negative result.

**Figure 4:**
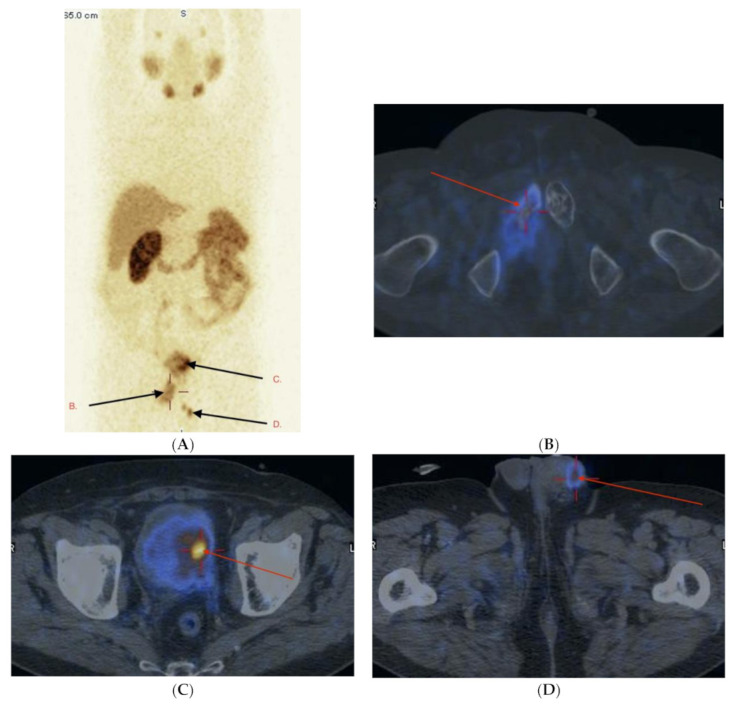
(**A**–**D**) Male 66 years, cTxN1M1, initially non-resectable, PCa, GlS = 7 (4 + 3). PSA 35.2 ng/mL. After palliative radiotherapy on the left hip and pubic bones, during hormonal therapy, previous history of hematuria. Currently, on the basis of TURCUT biopsy a bladder tumor can be seen on the left wall, also distal urethra involvement. Histology PCa GlS = 9 (4 + 5). Whole body WB-SPECT/CT (MIP) (**A**) and additional image fusion SPECT/CT (**B**–**D**) with [^99m^Tc]Tc-PSMA-T4. High pathological uptake of radiotracer seen in right pubic bone (arrow B), left bladder wall involvement (arrow C) and distal urethra involvement (arrow D).

Evaluation of PCa stage of disease in 10 subjects before surgery were confirmed by pelvic or obturator lymph nodes involvement; 9 of them had abdominal, chest or neck lymph nodes involvement. Local extension of primary PCa with local tumor infiltration of the bladder wall and separate foci of cancer in penis urethra, all confirmed by pathology after cystoscopic examination, were found and are presented in Figure 4A–D.

In two subjects with pathologic pelvic lymph node involvement, lesions were not detected in the [^99m^Tc]Tc-PSMA-T4 scan after surgery. One case with 2/5 lymph nodes involved had them removed during surgery, another subject had massive pulmonary emboli (PE) and locally advanced disease with an enlarged single external iliac lymph node seen in MRI, measuring 14 mm, which indicated a high probability of cancer involvement. This particular patient was discharged from surgery and switched to palliative therapy. In both cases, lymph nodes were not seen in the PSMA-T4 scans, despite high accumulation within the primary prostate tumor (grade of accumulation of radiotracer: 4/5).

**Figure 5:**
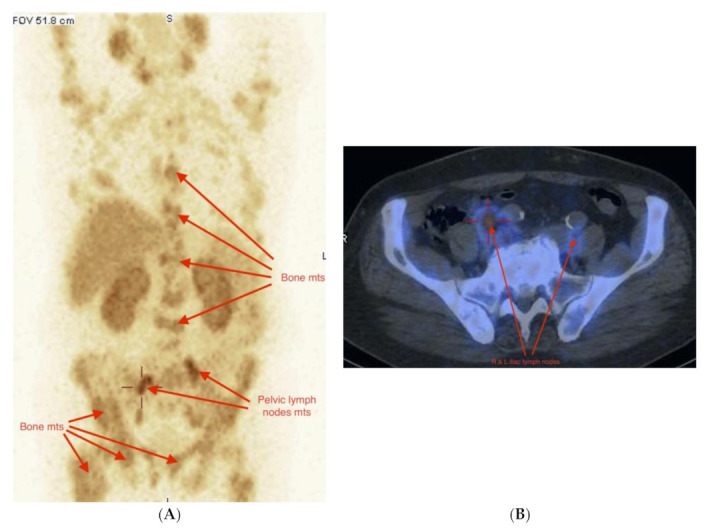
(**A**,**B**) Male 71 years, non-resectable PCa GlS = 9 (4 + 5), cTxN1M1, PSA = 7.2 ng/mL. During palliative hormonal and chemotherapy. Multiple bone mets and bilateral pelvic lymph nodes involvement in WB-SPECT/CT [^99m^Tc]Tc-PSMA-T4 evaluation of stage of disease. (**A**) WB-SPECT/CT, right and left enlarged pelvic lymph nodes and multiple bone mets in WB-SPECT (MIP) scan. (**B**) Osteosclerotic pelvic bone mets with pathological uptake of [^99m^Tc]Tc-PSMA-T4.

Considering the imaging of bone mets in this group of subjects, 10 of them had at least a single met, but most of them had multiple bone mets (Figure 5A,B).

Overall, 20 patients had true positive results, with confirmation of active pathology based on histopathology, clinical and imaging follow-up. One patient had a false negative result with no accumulation in PCa within the prostate gland. Two patients had no pelvic lymph nodes accumulation with pathological confirmation of PCa, but true positive focal pathological tumor accumulation in the prostate. Ten subjects had no focal uptake of radiotracer, confirmed with clinical, biochemical and imaging follow-up. In 6 cases, positive scans results had an influence on changing therapeutic decisions. The summarized results of the [^99m^Tc]Tc-PSMA-T4 examination for the whole group of patients included in the study are presented in Table A3.

### 2.5. Adverse Events (Safety)

[^99m^Tc]Tc-PSMA-T4 was well tolerated in every subject. In our study, we did not record any adverse events (AEs) or clinically detectable pharmacologic effects in any of the study subjects. There were also no changes in a physical examination in vital signs and also nothing significant in laboratory measurements and ECG.

## 3. Discussion

PSMA is one of the most promising biomarkers in subjects with PCa, which are currently successfully used in routine clinical practice. This is also the most attractive diagnostic and therapeutic approach (theranostic) that is currently under clinical investigation [2,3,5,8,16,17,18]. Most current efforts are concentrated on PET technology [8,9,10,11,12,19,20,21]. Several publications concerning PSMA-binding inhibitors, labeled initially with ^123^I and then ^99m^Tc, which contain a carbonyl system for radionuclide bonding in their structure were released over ten years ago [22,23,24,25]. However, the described preparations had some disadvantages, such as slow pharmacokinetics, high liver uptake, and slow clearance in the gastrointestinal tract, which can disrupt their application in prostate cancer (PC) imaging because this type of cancer most frequently metastasizes in the lower part of the spine, pelvis and lymph nodes within the abdomen. In recent years, many PSMA inhibitor analogues containing technetium-99m chelating systems of the HYNIC type have been created, the presence of which has significantly decreased the lipophilicity of the radiolabeled preparations [26,27,28,29,30,31,32,33,34]. To date, there are only a few publications that have indicated the potential clinical use of technetium-99m labeled PSMA [24,30,31,32]. Here, we have reported our initial clinical experience with the use of [^99m^Tc]Tc-PSMA-T4 radiolabeled ligand in subjects with prostate cancer, which was initially and recently described by Sergieva et al. [34,35]. The potential broader use of technetium (^99m^Tc) is suggested, because this radiotracer has the ideal nuclear properties of energy (140.5 keV photon) and half-life (6 h) and it is ideal for routine clinical use in all nuclear medicine departments equipped with standard a SPECT/CT gamma camera. Additional use of convenient commercial technetium generators offers the possibility of wide clinical use of ^99m^Tc labeled ligand any time of the working day of the nuclear medicine department [30,31,32,34,35].

The polyvalent nature of technetium, however, demands that due consideration is given to the chemical approach used for its incorporation into a targeting ligand. How the metal is bound can greatly influence the overall pharmacologic character and the biologic fate of the complex by altering the overall charge, polarity, and hydrophobicity.

The chelate, the metal–chelate complex, and the nature of the outer solvation sphere of the metal chelate complex all directly or indirectly influence the performance of such a radiolabeled ligand in regards to its intended clinical use [23,33]. Investigators at Molecular Insight Pharmaceuticals (MIP) developed a series of novel glutamateurea (glu-urea) amino acid heterodimeric inhibitors of PSMA for developing SPECT radiopharmaceuticals using ^123^I or ^99m^Tc [22,23,24]. However, the described technetium-99m labeled inhibitors, which contain a carbonyl system for radionuclide bonding in their structure have some disadvantages, as described above. On the other hand, according to the published biodistribution data for the developed preparations containing iPSMA and HYNIC chelator for technetium-99m [29,30], they exhibit very high accumulation in kidneys, which can cause difficulties in the interpretation of scintigraphic images. The iPSMA derivative used, e.g., in the iPSMA-HYNIC inhibitor contains one naphtylalanine (L-2NaI) linker only, which is likely to translate into the observed unfavorable pharmacokinetics of the compounds labeled with Technetium-99m. It has been clearly shown by Benesova et al. [36] that the pharmacokinetic properties, including PSMA inhibition potencies, cellular internalization, and the biodistribution behavior of the PSMA inhibitors can be significantly influenced by modification of the linker. Thus, the chemical constitution of the linker moiety also has a significant impact on in vivo tumor-targeting and the pharmacokinetic properties of PSMA-targeting radioligands.

Therefore, the PSMA-T4 has been developed considering the most suitable linkers set for Glu-urea-Lys to connect HYNIC moiety, which could lead to an improvement in the pharmacokinetics of the ^99m^Tc-PSMA compound by increasing tumor accumulation and reducing kidney accumulation. The studies undertaken showed that the presence of L-tryptophan (L-Trp) in PSMA-T4, as one of the linkers, instead of naphthylalanine (L-2NaI), led to a significant improvement in the biodistribution of the labeled molecule and its increased affinity to PSMA in vivo [33].

The new PSMA-T4 inhibitor, for ^99m^Tc radiolabeling, was developed by the inventors of POLATOM in the form of a single vial radiopharmaceutical kit. This is the most convenient way of obtaining a ready-to-use diagnostic preparation in clinical conditions. The sterile, single vial kit provides a simplified labeling procedure consisting of only two stages: the addition of sterile pertechnetate (^99m^Tc) eluate from a ^99^Mo/^99m^Tc generator and incubation at 95 °C for 15 min leads to a ready-to-use radiopharmaceutical of high radiochemical purity (RCP > 95%) [33].

In our pilot study, we randomly selected 31 patients with different intermediate and high risk PCa in terms of the biology of cancer (Gleason score), a stage of disease localized in subjects with an initial diagnosis, and those with extensive disease based on clinic and imaging reports. Additionally, those with recurrence after different therapy approaches such as radical prostatectomy or external beam radiotherapy were selected. Similar to others, we found that delayed images have higher accuracy compared to early images [30,31,34,35]. A great benefit of delayed images is the gradual accumulation of radiotracer within target lesions with no degradation in image quality, particularly in tomographic SPECT images, which is seen with other receptor binding radioligands in somatostatin receptor scintigraphy (SRS) using [^99m^Tc]Tc-HYNIC-TOC or [^99m^Tc]Tc-HYNIC-TATE. The high quality of images was seen in all patients with the active disease based on three points of analysis of SUVmax (lbm), see Figure 1. Our results indicated that this radio-compound could be used in different clinical scenarios for initial diagnosis, recurrence and re-staging of disease as an additional study of different biologically PCa behavior, independent of low or high Gleason scores. Only one patient with confirmed PCa in the prostate gland had a negative scan, which confirms the earlier observation of some PSMA-negative uptake prostate cancer lesions. We also documented a false positive result due to the presence of advanced lung carcinoma. Despite relatively weak accumulation within lung carcinoma, it could be suggested the [^99m^Tc]Tc-PSMA-T4 is not highly specific for PCa cells. Three patients suspected of recurrence after radical radiotherapy had true negative results in the prostate (which requires further observation), but two had bone mets, which were positive in [^99m^Tc]Tc-PSMA-T4 scans.

Patients with local and regional lymph nodes disease were correctly evaluated in 83% of cases. Two patients had a false negative scan of disease present within lymph nodes with no clear explanation of the absence of accumulation of radiotracer, as in both cases there was high accumulation in primary cancer in the prostate. High accuracy (sensitivity and specificity) was found in the detection of extra pelvic lymph nodes and bone mets. One patient with GlS = 7 had an iliac 14 mm lymph node and also had 4 of 15 lymph nodes involved, and none were positive in the [^99m^Tc]Tc-PSMA-T4 scan. The patient had surgery 11 weeks after the scan, which could potentially explain the difference in the final pathological results. In 3 cases of positive scans with equivocal PSA as a biochemical recurrence, the positive scan implied a change in treatment. This finding suggests that [^99m^Tc]Tc-PSMA-T4 may have the potential to find disease progression earlier than the standard bone scan. In addition, this approach seems to be more sensitive in the detection of lymph node disease, even in those measuring less than 8 mm, which is the normal size threshold criteria based on CT or MRI [37]. This is also suggested by others, that is, with the high sensitivity of ^99m^Tc-labeled radioligands we can improve the detection of N1 disease and possibly help to guide the extended surgical resection so that the correct evaluation of cancer extent is the principal task of examination [24,30,31,34,35].

The disadvantage of our pilot study was the relatively small number of subjects, so the results need to be verified in a prospective multicenter phase II trial with a larger group of patients with intermediate and high risk PCa. Another drawback is the difficulty of confirming that lesions indicated by PSMA are really PCa lesions; however, but this is similar to reports on other imaging modalities, which investigated clinically advanced stages of disease with no possibility of verifying all detected lesions [38].

The important advantages of the use of [^99m^Tc]Tc-PSMA-T4 with SPECT are the relatively low cost of the examination and use of the standard SPECT/CT approach, which is more suitable in comparison to PET installation in most nuclear medicine departments. Additionally, there is a risk that in the high population cohort of men with suspected or diagnosed prostate cancer who require PSMA imaging, many may have significantly delayed PSMA imaging with PET technology due to insufficient PET facilities, and the inability to perform the scan quickly also creates potential problems in further diagnostic and therapeutic optimal approach. Therefore, it seems that the clinical use of [^99m^Tc]Tc-PSMA-T4 may be an alternative for accelerating the correct diagnosis and may be helpful in selecting patients for a more precise assessment with the use of [^68^Ga]Ga PSMA-11 PET in inconclusive cases of WB-SPECT/CT examination.

## 4. Materials and Methods

This was a pilot, prospective, interventional, single institution, preliminary case series study, which was approved by the Clinical Ethics Committee University of Warmia and Mazury, Olsztyn; Poland No. 5/2019. Prior to inclusion in the study, all patients understood the experimental nature of the imaging diagnosis and all of them signed their written informed consent form.

The study group consisted of 31 patients with pathological confirmation of PCa. The indications for scanning included an evaluation of the tumor stage before planned radical prostatectomy in 3 cases, 8 patients had advanced stage of disease and had an evaluation of disease extent and metabolic activity based on [^99m^Tc]Tc-PSMA-T4 examination. The rest of the patients (20) had clinical or biochemical suspicion of cancer recurrence. 

The other inclusion criteria of the study were: staging of increased PSA level after initial biopsy or increasing PSA level after radical treatment in patients with active clinical and biochemical follow-up and willingness to participate in this study and to provide written informed consent (see Supplementary Materials). The demographic and clinical data for all patients included in the study are presented in Table A1. The summarized clinical, pathological and biochemical data are presented in Table A2.

### 4.1. Study Drug

The PSMA-T4 (Glu-CO-Lys-L-Trp-4-Amc-HYNIC) (C_37_H_49_N_9_O_10_; MW 779.36 g/mol) ligand was designed and synthesized at POLATOM (National Centre for Nuclear Research, Radioisotope Centre POLATOM; Polish patent P.429630, EP19199838.4 [29] and US 16559014 (patents pending). The details are shown in the Supplementary Materials.

#### 4.1.1. PSMA-T4 Freeze-Dried Kit for Radiopharmaceutical Preparation

The pharmaceutical kit of PSMA-T4 for labelling with Technetium-99m eluate was developed for direct and simple preparation of [^99m^Tc]Tc-PSMA-T4 radioligand and is sterile and endotoxin free. The injection can be directly applied in patients without the need for any further purification process. The dry composition comprises PSMA-T4 inhibitor, reducing agent SnCl_2_ × 2H_2_O, co-ligands for obtaining a stable complex with radiometal: tricine and ethylenediamine-(N,N′)-diacetic acid (EDDA), as well as phosphate buffer for pH adjustment. The freeze-dried kit is prepared under GMP conditions, and the content is protected from oxidation by the inert atmosphere of sterile nitrogen.

#### 4.1.2. Preparation of [^99m^Tc]Tc-PSMA-T4 and Evaluation of Radiochemical Purity

A total of 1.0–2.5 mL of sodium pertechnetate Na[^99m^Tc]TcO_4_ solution from a ^99^Mo/^99m^Tc generator (PolGentec, POLATOM) with the required radioactivity (370–1000 MBq) was introduced to the vial containing the dry composition from the PSMA-T4 kit. After dissolving, the solution was heated at 95 °C for 15 min and then cooled down at room temperature for another 20 min. The radiochemical purity of the preparation obtained in such a way was assessed by thin layer chromatography and was above 95% in each case.

### 4.2. Image Acquisition

All patients underwent [^99m^Tc]Tc-PSMA-T4 SPECT/CT examinations, 20 subjects were initially assessed three times at various time points as follows: 1–1.5 h, 3–4 h and 6–7 h. The rest of the patients were subject to a single acquisition between 4–6 h. All patients were scanned after i.v. injection with mean activity of 436 MBq (range 310–540 MBq) of [^99m^Tc]Tc-PSMA-T4. In each case, whole body WB-SPECT-CT was performed using a gamma camera NM/CT 860 (GE, Healthcare, Milwaukee, WI, USA). Details are presented in the Supplementary Materials.

### 4.3. Image Analysis

An evaluation of each study was performed based on an independent visual interpretation by a nuclear medicine physician (JBC), who was experienced in the interpretation of receptor studies using technetium labeled radiopharmaceuticals. The reader of the scans was aware of the underlying pathology and the results of the standard staging procedures. The corresponding studies were compared and analyzed lesion by lesion.

Any focal tracer accumulation that exceeded the expected regional tracer uptake was rated as a pathologic finding (tumor uptake). Diffuse high activity intestinal uptake as well as liver, kidney, spleen, salivary and lacrimal glands were rated as non-specific, physiologic uptake.

The detection rate of the pathological uptake of radiotracer (RT) in each patient was evaluated by qualitative visual analysis and then calculated as semi-quantitative results as follows: score 1: no abnormal uptake of radiotracer—no cancer; score 2: low, diffuse increased uptake—low probability of cancer; score 3: low or moderate increased of uptake, in focal shape—equivocal results; score 4: high increased uptake—probability of cancer; score 5: very high uptake of radiotracer—definitive cancer. All data were analyzed from WB-SPECT/CT images using an Xeleris 4DR Workstation (GE Healthcare, Milwaukee, WI, USA).

Ten subjects with an active pathology were subject to additional analysis using a volume-of-interest (VOI), which were deposited over the most active regions of the tumor/tumors for quantitative analysis. The VOIs were placed to avoid interference with other structures with high physiological accumulation of radiotracer. The VOIs were verified and measured in three dimensions using standard planes as axial, coronal and sagittal views. The size of the VOI in each case was at least 15 mm in diameter. For quantitative data regarding the radiotracer accumulation, we used the data sets of 10 patients with active pathologies including 10 VOIs placed on the primary prostate cancer, 7 placed in the involved lymph node and another 8 were placed in selected bone mets. Overall, 25 pathological lesions were used to analyze the activity in 3 different time frame acquisitions. Details are provided in the Supplementary Materials.

### 4.4. Adverse Events (Safety)

Potential adverse events (AEs) were recorded during the examination and for 24 h after injection of the radiopharmaceutical. The physical examination of vital signs and also laboratory measurements (including clinical chemistry, hematology) were documented. The safety analysis was performed based on data from all subjects. Adverse events were recorded based on CTC-AEs (National Cancer Institute Common Terminology Criteria) ver. 5.0.

### 4.5. Statistics

In this analysis, all data are presented as the mean (range and /or SD, SEM). The comparison of the quantitative variables for the sets of images with high active radiotracer uptake in tumors was drawn manually, and ROIs were based on the value of SUVmax (lbm). The analysis was done using the Wilcoxon paired match test. P < 0.05 was considered statistically significant in all comparisons (Statistica v.13.3; TIBCO Software Inc. Palo Alto, CA, USA).

## 5. Conclusions

[^99m^Tc]Tc-PSMA-T4 WB-SPECT/CT is a cost-effective diagnostic tool in patients with PCa, which may benefit those with an initial diagnosis of PC, those who have an evaluation of the clinical stage of disease and those in whom recurrent disease is suspected. This could be used as an alternative to PET technology, particularly in the setting of nuclear medicine departments without a PET facility. This approach seems to be quite effective in primary tumor detection but is probably even more efficient in lymph nodes and bone metastatic disease. The presented case series provides proof-of-concept findings that need to be further investigated in the future in a larger number of patients.

## Data Availability

The data presented in this study are available on request from the corresponding author. The data are not publicly available due to a pilot trial, performed as prospective, interventional, single institution, preliminary case series study, which was approved by the Clinical Ethics Committee University of Warmia and Mazury Olsztyn Poland. The next step of trial as multicenter study will be available on www.clinicaltrials.gov, accessed on 20 October 2021.

## Supplementary Materials

The following are available online at https://www.mdpi.com/article/10.3390/ph14111107/s1.

## Appendix A

**Table A1:** Demographic and clinical data of all patients included into the study including Gleason score, radical or palliative initial therapy, pathological or clinical diagnosis, TNM classification PSA level before PSMA examination.

Pt No	Age	Indication	GlS **	R/P #	Primary Diagnosis	TNM $	PSA before Scan (ng/mL)	Doubling Time PSA (Months)
1	53	Stage	4 + 5	P	21.01.2014	cTxN1M1	4.38	n.d.
2	65	Rec	5 + 3	R	01.02.2014	pT3bN0Mx	0.1	>12.0
3	66	Stage	3 + 4	P	01.06.2014	cTxN1M1	35.2	
4	65	Rec	4 + 3	R	12.04.2018	pT3bN0Mx	10.4	1.2
5	67	Rec	4 + 3	R	02.03.2018	pT3aN0M0	1.41	n.d.
6	66	Rec	5 + 4	R	11.05.2016	pT3bN0Mx	1.73	4.7
7	78	Stage	5 + 5	P	27.03.2019	cTxNxM1	10.2	
8	72	Rec	3 + 4	R	22.12.2015	pT2aN0M0	1.54	2.0
9	56	Stage *	4 + 4	R	30.04.2019	pT3bN1Mx	25.9	
10	69	Stage	4 + 3	P	13.04.2018	cT4N1M1	54.8	n.d.
11	72	Rec	4 + 3	R	30.11.2018	pT2cN0Mx	0.48	n.d.
12	76	Rec	4 + 5	R(Rtx)	23.05.2013	cT2cNxM0	0.28	>12.0
13	66	Stage	4 + 5	P	02.02.2012	cTxNxM1	0.35	n.d.
14	59	Rec	3 + 3	R(Rtx)	04.06.2008	cT2cNxM0	0.84	>12.0
15	69	Rec	3 + 4	R	30.07.2019	pT3bN1Mx	9.39	4.1
16	68	Rec	3 + 4	R	17.10.2018	pT3bN1Mx,	0.1	
17	63	Rec	3 + 5	R	26.02.2013	pT3cN1Mx	5.99	n.d.
18	69	Rec	3 + 5	R	01.09.2017	pT3bN0M0	5.68	10.0
19	68	Rec	4 + 3	R	08.04.2019	pT2bN0Mx	0.23	
20	81	Stage	3 + 3	P	18.04.2018	cTxNxM1	29.6	>12.0
21	67	Rec	4 + 3	R	25.05.2018	pT3bN1Mx	12.4	n.d.
22	71	Stage	4 + 5	P	29.03.2019	cTxN1M1	7.2	
23	74	Rec	4 + 3	R	20.11.2018	pT3bN0M0	0.2	n.d.
24	66	Stage *	4 + 3	P	29.05.2019	cT3bN1Mx	6.94	>12.0
25	66	Rec	3 + 4	R	10.03.2015	pT2aN0M0	1.6	n.d.
26	62	Rec	3 + 4	R	02.04.2014	pT2CN0M0	0.12	9.0
27	87	Stage	4 + 4	P	18.03.2019	cTxNxM1	228.0	>12.0
28	59	Rec	3 + 3	R	03.08.2005	pT2cN0Mx	3.3	3.0
29	69	Rec	5 + 5	R	20.12.2018	pT3bN1Mx	0.14	
30	66	Rec	3 + 4	R	10.06.2016	pT2cN0Mx	0.02	5.0
31	76	Stage *	4 + 3	R	01.02.2019	pT3bN0Mx	6.63	>12.0

Indication of PSMA scanning Stage: evaluation of disease extent and metabolic activity (before radical therapy * or palliative settings); Rec: evaluation of recurrence after radical therapy; GlS **: Gleason Score; R/P #: Radical or Palliative Therapy, previous or current; TNM $: pathologi-cal pTNM or clinical cTNM classification of PCa; R(Rtx): radical radiotherapy.

**Table A2:** Summarized clinical, pathology and biochemical data for the group of patients with PCa included in the study.

Therapy	Number of Subjects *n* (%)
Radical prostatectomy	22 (71)
Palliative treatment including different combination of radiotherapy, chemotherapy, hormonotherapy	9 (29)
Radical surgery (Intention to Treat–ITT)	20 (65)
R0	10 (50)
R1/R2	10/0 (50/0)
Radical Radiotherapy (Rtx)	2 (10)
**Gleason Score (GlS) *n* = 31**	Number of subjects *n* (%)
GLS = 6 (3 + 3)	3 (10)
GLS = 7 (3 + 4 or 4 + 3)	16 (52)
GLS = 8 (3 + 5; 4 + 4 or 5 + 3)	5 (16)
GlS = 9 (4 + 5 or 5 + 4)	5 (16)
GlS = 10 (5 + 5)	2 (6)
**Risk group**	Number of subjects *n* (%)
Intermediate	19 (62)
High	12 (32)
**Indication to Scanning *n* (%)**	Number of subjects *n* (%)
Evaluation of disease extent—palliative settings	8 (25)
Evaluation of disease extent—before radical therapy	3 (10)
Evaluation of Recurrence after radical therapy	20 (65)
**PSA at imaging (mean, range)**	Mean (range)
Radical therapy	3.34 (0.02–12.4)
Palliative	52.3 (7.2–228)

**Table A3:** Summarized results of [^99m^Tc]Tc-PSMA-T4 examination for the whole group of patients included in the study.

Target are of Interpretation of Results [^99m^Tc]Tc-PSMA-T4 Scan	True Positive *n*: Sensitivity (%)	True Negative *n*; Specificity (%)	False Negative *n* Negative Predictive Value (%)	False Positive *n*; Positive Predictive Value (%)
Prostate (*n* = 15)	11 (92)	3 (100)	1 (75)	0 (100)
Pelvic lymph nodes (per patient)	10 (83)	19 (100)	2 (91)	0 (100)
Abdominal/thoracic/other lymph nodes or soft tissue involvement (per patient)	9 (100)	21 (95)	0 (100)	1 (90)
Bone mts (per patient)	11 (100)	20 (100)	0 (100)	0 (100)
