# Supplementary material for: Initial Experience of Clinical Use of [99mTc]Tc-PSMA-T4 in Patients with Prostate Cancer. A Pilot Study
Source: Pharmaceuticals (Basel). 2021 Oct 29;14(11):1107. doi: 10.3390/ph14111107 (PMC8623387; doi:10.3390/ph14111107)
Supplement: Supplementary file 1 [file pharmaceuticals-14-01107-s001.zip › JBCwikla-PSMA-T4-informed-consent-form.pdf]

Ver. 1.0 z dn. 07.12.2018

INFORMACJA DLA PACJENTA

Tytuł badania:

„Diagnostyka przerzutów raka prostaty technikami SPECT i WB z wykorzystaniem ^{99m}Tc-PSMA-T4"

**Lekarz prowadzący**	**Dr hab. n. med. Jarosław B. Ćwikła Prof. UWM** – Katedra Kardiologii i Kardiochirurgii; Collegium Medicum, Wydział Nauk Lekarskich; UWM w Olsztynie; Warszawska 30; 10-082 Olsztyn
**Podmiot Prowadzący:**	Pracownia Medycyny Nuklearnej; Katedra Kardiologii i Kardiochirurgii; Collegium Medicum, Wydział Nauk Lekarskich; UWM w Olsztynie; Warszawska 30; 10-082 Olsztyn
**Lokalizacja Badania**	**Pracownia Medycyny Nuklearnej; Katedra Kardiologii i Kardiochirurgii;** Wydział Lekarski, Collegium Medicum, Uniwersytecki Szpital Kliniczny (USK) w Olsztynie; Warszawska 30; 10-082 Olsztyn<br><br>**Centrum Diagnostyczno-Lecznicze „Gammed"** 02-351 Warszawa, Lelechowska 5; tel. 228223001; 507089942; fax 228220315 gammed@gammed.pl; www.gammed.pl
**Sponsor Badania**	**Brak -** nieodpłatne udostępnienie preparatu badanego przez NCBJ Polatom, Otwock

Część 1

Zapraszamy Pana do udziału w badaniu klinicznym w celu diagnostyki Pana schorzenia onkologicznego. Zespół diagnostyczno-terapeutyczny w tej placówce zajmuje się między innymi zagadnieniami diagnostyki raka stercza choroby, która została u Pana rozpoznana. Lekarz prowadzący badanie wyjaśni Panu/Pani, na czym polega obecny eksperyment medyczny, do którego Pana zapraszamy. Udział w nim biorą wyłącznie osoby, które wyrażą na to zgodę. Aby móc podjąć świadomą decyzję o udziale, należy dokładnie zapoznać się jakie są korzyści oraz jakie zagrożenia wiążą się z udziałem w tym eksperymencie medycznym. Prosimy uważnie przeczytać podane tu informacje i w razie potrzeby omówić je z rodziną, przyjaciółmi i lekarzem pierwszego kontaktu (lekarzem rodzinnym) lub lekarzem prowadzącym dotychczasowe leczenie. Prosimy bez pośpiechu zastanowić się nad decyzją o udziale. W razie dalszych pytań można też poprosić lekarza prowadzącego badanie o dodatkowe wyjaśnienia.

Część 2 informuje, w jakim celu badanie jest prowadzone i na czym polega w nim udział.

1. W JAKIM CELU PROWADZONE JEST TO BADANIE?

Ten eksperyment medyczny jest badaniem tzw. własnym (akademickim) prowadzonym przez lekarza medycyny nuklearnej dr hab. N. Med. Jarosław Bogdan Ćwikła prof. UWM w Olsztynie. Badanie prowadzone jest we współpracy z podmiotem wytwarzającym badany produkt medyczny Narodowe Centrum Badań Jądrowych, (NCBJ) Ośrodek Radioizotopów, Polatom, Otwock.

Projekt nie jest jednak typowym badaniem komercyjnym sponsorowanym przez zewnętrzny podmiot, jest to eksperyment medyczny celem w przetestowania klinicznego nowej substancji do badania chorych na raka stercza. Jest to tym samym autorski projekt Collegium Medicum oraz NCBJ z Ośrodkiem Radioizotopów, Polatom. Faza badana eksperymentu medycznego po jej zakończeniu i analizie wyników da podstawy do zorganizowania badania klinicznego celem rutynowego wprowadzenia badanego produktu leczniczego do praktyki klinicznej.

Głównym celem prowadzonego eksperymentu medycznego będzie przetestowanie klinicznej użyteczności produktu medycznego ^{99m}Tc PSMA-T4 u chorych z patologicznym rozpoznaniem raka stercza, w celu potwierdzenia jego obecności wraz z oceną stadium zaawansowania na podstawie badania scyntygraficznego prowadzonego za pomocą techniki tomograficznej SPECT lub SPECT/CT oraz techniki badania całego ciała (WB – whole body).

2. INFORMACJE DLA PACJENTA ZAWIERAJĄ OMÓWIENIE BADANIA

Projekt obejmuje udział w eksperymencie, w którym to podamy dożylnie nową obiecującą substancję wyznakowaną radioizotopem technetu w celu diagnostyki stwierdzonej u Pana choroby (raka stercza/prostaty). W tej fazie badania zostanie poddanych badaniu 10 chorych mężczyzn z rozpoznaniem raka stercza.

3. DLACZEGO ZAPROSZONO MNIE DO BADANIA?

Zapraszamy Pana do udziału w tym projekcie, ponieważ stwierdzono u Pana raka prostaty i Pana lekarz prowadzący wskazał na rozszerzenie diagnostyki obrazowej o inne metody diagnostyczne obejmujące między innymi techniki czynnościowe, w tym radioizotopowe. Obecnie w badaniach radioizotopowych w kierunku obecności procesu chorobowego o typie raka stercza wykorzystuje się technikę PET w oparciu o znaczniki wykorzystujące radioizotop galu (^{68}Ga PSMA-11) czy węgla (^{11}C-cholina). Badania te są jednak drogie oraz ogólnie stosunkowo mało dostępne, dysponują nimi tylko pojedyncze placówki w kraju.

Dlatego alternatywnie z uwagi na większą dostępność w pracowniach medycyny nuklearnej urządzeń o gamma kamera z obrazowaniem tomograficznym SPECT, które to bazuje na podobnych pryncypiach obrazowania tomograficznego jak PET. Jednocześnie chcemy zaproponować udział w eksperymencie medycznym z wykorzystaniem radioizotopu technetu (99mTc) oraz z wykorzystaniem gamma kamery SPECT. Badania będę wykonywane po podaniu dożylnym wyznakowanego technetem (99mTc) preparatu o nazwie PSMA-T4, który selektywnie gromadzi się na komórkach raka stercza.

Tym samym informujemy Państwa, że istnieje możliwość zastosowania relatywnie nowego typu diagnostyki czynnościowej Pana choroby w którym testowany produkt medyczny może być potencjalnie pomocny w prawidłowej ocenie obecności raka wraz z oceną stadium zaawansowania procesu chorobowego, celem określenia dalszego postępowania na podstawie stosunkowo prostego i w przyszłości bardziej dostępnego badania scyntygraficznego w porównaniu do techniki PET.

W eksperymencie opisanym powyżej będzie uczestniczyło do 10 chorych. W badaniu tym zostanie określony poziom gromadzenia radioznacznika w rzucie potencjalnych lub wcześniej wykrytych zmian chorobowych, będącymi ogniskami pierwotnymi i/lub przerzutowymi raka.

Badanie to jest otwarte tzn. każdy chorych może brać udział w badaniu po spełnieniu wszystkich kryteriów włączenia i niespełnieniu kryteriów wyłączenia. Badanie będzie prowadzone równolegle w 2 ośrodkach nadzorowanych przez kierownika badania będącego jednocześnie lekarzem nadzorującym badania w Olsztynie w Pracowni medycyny nuklearnej, zlokalizowanej w Uniwersyteckim Szpitalu Klinicznym, 10-082 Olsztyn, Warszawska 30 oraz alternatywnie w Centrum Diagnostyczno-Leczniczym Gammed; 02-351 Warszawa, Lelechowska 5.

4. CZY MUSZĘ BRAĆ UDZIAŁ W BADANIU?

Decyzja o udziale w badaniu jest dobrowolna. Lekarz prowadzący badanie wyjaśni Panu/Pani, na czym polega badanie i omówi z Panem treść tych informacji. Jeżeli zdecyduje się Pan/Pani na udział, poprosimy Pana o podpisanie formularza świadomej zgody, a niniejszy dokument informacji dla pacjenta otrzyma Pan na własny użytek.

Pacjent, który zdecyduje się na udział w badaniu może je w każdej chwili opuścić. Nie trzeba podawać przyczyny, a decyzja o opuszczeniu badania nie wpłynie ujemnie na standard otrzymywanej opieki medycznej - obecnie lub w przyszłości. Jeżeli nie zdecyduje się Pan na udział, będzie Pan nadal otrzymywać najlepsze leczenie w Pana przypadku, dostępne w ramach obowiązującego systemu opieki zdrowotnej.

5. JAKIM BADANIOM ZOSTANĘ PODDANY?

Badanie polegać będzie na podaniu dożylnym diagnostycznej dawki ^{99m}Tc PSMA-T4 w postaci pojedynczego wstrzyknięcia dożylnego przygotowanego w pracowni medycyny nuklearnej radioizotopowego preparatu diagnostycznego. Podawany radiofarmaceutyk będzie w objętości ok. 5ml roztworu, o aktywności ok. 740MBq radioizotopu ^{99m}Tc. Jest to standardowa dawka radioizotopu, która jest powszechnie podawana w przypadku wszelkich innych badań jak scyntygrafia kości, badanie scyntygraficzne serca, badanie receptorów somatostatynowych i wiele innych tego typu procedur medycyny nuklearnej. Bezpośrednio po podaniu dożylnym radiofarmaceutyku wenflon przez który odbywało się podanie zostanie przepłukany roztworem soli fizjologicznej w ilości ok. 20ml. Jak wspomniano powyżej podana dawka radioizotopu jest powszechnie akceptowaną dawką diagnostyczną wykorzystywaną rutynowo w badaniach medycyny nuklearnej. Wiązanie tego znakowanego ligandu, którym jest badana substancja o nazwie ^{99m}Tc PSMA-T4, to jak wykazały badania laboratoryjne oraz wstępne badania kliniczne jest trwałe i swoiste na komórkach raka stercza na których stwierdzona jest nadekspresja receptora dla PSMA.

Badanie będzie wykonywane w ramach diagnostyki choroby, na którą Pan choruje na zlecenie lekarza prowadzącego, najczęściej urologa i/lub onkologa klinicznego, czy radioterapeuty. Cała procedura badawcza w ramach prowadzonego eksperymenty medycznego prowadzona jest w trybie ambulatoryjnym. Z uwagi na charakter badawczy w którym Pan potencjalnie będzie brał udział, będziemy prosili o Pana pomoc oraz cierpliwość związane z tym przeprowadzeniem wszystkich procedur związanych z tym badaniem. Cała procedura od podania radioznacznika dożylnie do momentu opuszczenia pracowni medycyny nuklearnej może trwać do 12h, dlatego prosimy o zarezerwowanie sobie całego dnia przy wyrażeniu przez Pana zgody na udział w tym badaniu.

6. OPIS PROCEDUR WYMAGANYCH W RAMACH BADANIA 99mTc PSMA-T4

Planowane badanie będzie odbywało się w Pracowni Medycyny Nuklearnej Uniwersyteckiego Szpitala Klinicznego w Olsztynie oraz alternatywnie w Centrum Diagnostyczno-Leczniczym „Gammed" w Warszawie i nadzorowane będzie przez doświadczonego lekarza specjalistę medycyny nuklearnej wraz z zespołem.

Chorzy proszeni będą o zgłaszanie się do konsultującego lekarza medycyny nuklearnej od kierującego urologa, onkologa czy radioterapeuty, po kwalifikacji wstępnej gdzie szczegółowo zostaną analizowane wszystkie kryteria włączenia i wyłączenia z badania i dokładnym wyjaśnieniu istoty badania choremu przez lekarza prowadzącego badanie i po uzyskaniu świadomej zgody na przeprowadzenie tego badania zostanie Pan zakwalifikowany do badania.

W ramach prowadzonego eksperymentu medycznego testowany będzie preparat radiofarmaceutyczny w którym po podaniu będą wykonywana cykliczne pobrania krwi oraz 3 obrazowania z wykorzystaniem standardowej gamma kamery SPECT/CT. Bezpośrednio po podaniu radiofarmaceutyku będzie Pan proszony o nawodnienie się poprzez przyjęcie płynów doustnie, najczęściej wykorzystywana jest woda niegazowana, ale mogą to być również inne płyny obojętne w zależności od Pana chęci.

Następnie po 30 min od podania radiofarmaceutyku zostanie Panu pobrana krew w ilości ok. 5ml, wykonana z żyły przeciwnej niż ta do której został podany preparat radioizotopowy. To pobranie ma na celu ocenę radioaktywności pozostającej w obrębie krwi obwodowej i ma znaczenie w ocenie dozymetrycznej. Następnie podobne pobranie krwi obwodowej będzie po ok. 1h od podania radioznacznika. To pobranie będzie bezpośrednio przed obrazowaniem Pana, które zostanie wykonane za pomocą standardowego urządzenia gamma kamera SPECT z wykorzystaniem techniki tomograficznej WB-SPECT/CT oraz dodatkowo w technice całego ciała (WB- whole body). Całość obrazowania będzie trwała około 60 min i jest to najdłuższe obrazowanie w ramach tej procedury.

Następnym elementem będzie kolejne 2 pobranie krwi obwodowej, tak jak poprzednio ok. 5ml, które zostanie wykonane po ok. 3-4 h od podania radioznacznika. Bezpośrednio po pobraniu krwi obwodowej zostanie Pan poproszony na ponowne badanie obrazowe wykonane tym razem tylko za pomocą techniki tomograficznej WB-SPECT/CT. Badanie będzie trwało ok. 40-45min. W ostatniej fazie badania będzie Pan proszony ponownie na pobranie krwi i będzie to pomiędzy 6-9h od podania radioznacznika. Bezpośrednio po pobraniu krwi, zostanie Pan poproszony na ponowne ostatnie już obrazowanie w tej samej technice WB-SPECT/CT, badanie to jak poprzednio będzie trwało ok. 40-45min. Po tym badaniu będzie Pan oceniony klinicznie przez lekarza prowadzącego badanie w celu oceny ewentualnych wczesnych objawów niepożądanych (AEs) oraz zostanie dokonany pomiar dozymetrii zewnętrznej, jaka wielkość promieniowania zostaje emitowana zanim opuści Pan pracownię Medycyny Nuklearnej.

Każdorazowo bezpośrednio przed obrazowaniem, czyli po 1 godz. od podania radioznacznika, następnie przed drugim obrazowaniem, czyli 3-4 godz. po podaniu radioznacznika oraz na końcu przed trzecim obrazowaniem pomiędzy 6-9 h od podania radioznacznika, zostanie Pan poproszony o oddanie moczu w toalecie, gdzie będziemy prosili Pana o oddanie ok. 50ml moczu ze środkowego strumienia do plastikowego pojemnika na mocz. Mocz ten podobnie jak Pana wcześniej pobrana krew pobierana przed obrazowaniem, będzie użyta do oceny dozymetrii wewnętrznej w celu oceny wielkości promieniowania w obrębie Pana narządów wewnętrznych.

Ostatnie pobranie krwi w zależności od Pana decyzji zostanie wykonane bezpośrednio przed opuszczeniem pracowni po dokonaniu pomiarów dozymetrycznych.

7. CZEGO MOGĘ SIĘ SPODZIEWAĆ, JEŚLI WEZMĘ UDZIAŁ W BADANIU?

Jeżeli zdecyduje się Pan na udział w proponowanym eksperymencie zostaną najpierw przeprowadzone tzw. badania wstępne, czyli różne testy w celu ustalenia, czy kwalifikuje się Pan do udziału w w/w eksperymencie.

Udział w badaniu jest oczywiście dobrowolny, ponieważ jest to badanie diagnostyczne monitorowanie Pana będzie trwało przez cały okres przebywania Pana w Pracowni Medycyny Nuklearnej. Następnie z uwagi na udział w eksperymencie medycznych będziemy z Panem w kontakcie przez następny tydzień w celu oceny potencjalnych późnych działań niepożądanych które potencjalnie, jeśli wystąpią będą odpowiednio raportowane.

Należy pamiętać, że zgoda na udział w proponowanym badaniu oznacza, że nie można w tym samym czasie uczestniczyć w innym podobnym badaniu klinicznym z wykorzystaniem podobnych substancji lub innych substancji znacząc ingerujących w ten projekt. Dopuszczalny jest udział w badaniu obserwacyjnym oraz badaniu jakości życia w ramach EROTC.

A. <u>Przed rozpoczęciem udziału w badaniu, a po podpisaniu świadomej zgody na udział w badaniu zostanie sprawdzone:</u>

- Badanie wstępne podczas tej wizyty lekarz prowadzący badanie dokona pomiaru parametrów życiowych (ciśnienia krwi, tętna, masy ciała i wzrostu) oraz przeprowadzi pełne badanie lekarskie (tzw. badanie fizykalne). Zapyta o Pana/Pani ogólny stan zdrowia i zdolność wykonywania codziennych czynności, dokona oceny tzw. sprawności fizycznej chorego (PS). Zostanie wykonany zapis elektrycznej czynności serca (EKG) oraz zostaną odnotowane wszelkie leki przyjmowane obecnie jak i w przeszłości. Zostaną przeanalizowane wcześniej wykonane rutynowe badania krwi w celu oceny czynności nerek, wątroby i szpiku kostnego, akceptowalne będą wyniki badań laboratoryjnych wykonane poniżej 1 m-ca czasu.

B. <u>Lekarz prowadzący badanie oceni wcześniej wykonane badania i testy diagnostyczne:</u>

- Badania obrazowe obejmujące badania endoskopowe, badania strukturalne jak CT, MRI, USG inne jak również dostępne badania scyntygraficzne, czy PET z użyciem ^{68}Ga PSMA-11 czy 11C Cholina. Jeżeli którekolwiek z tych badań było u Pana/Pani wykonane w ciągu 6 miesięcy poprzedzających obecnie prowadzone badania będziemy prosili Pana o dostarczenie wyniku oraz badania na nośniku w formacie DICOM-3, w celu wspólnej analizy proponowanego eksperymentu medycznego oraz w/w badań obrazowych zarówno strukturalnych jak i czynnościowych.
- Wizyta kwalifikująca: podczas tej wizyty lekarz prowadzący badanie ostatecznie stwierdzi, czy kwalifikuje się Pan/Pani do udziału w proponowanym badaniu. Podczas tej wizyty i przed przeprowadzeniem jakiejkolwiek oceny poprosimy o wypełnienie kwestionariusza z pytaniami dotyczącymi samopoczucia i stopnia, w jakim choroba wpływa na funkcjonowanie w życiu codziennym, jest to formularz jakości życia EORTC dotyczący raka stercza. Następnie przeprowadzimy pełne badanie fizykalne oraz ocenę ogólnego stanu zdrowia i zdolności funkcjonowania w życiu codziennym, zapiszemy stosowane obecnie leki lub terapie oraz dokonamy pomiaru parametrów życiowych (masy ciała, ciśnienia krwi i tętna).

8. WYNAGRODZENIE

Nie otrzyma Pani wynagrodzenia za udział w badaniu. Ponieważ nie jest to projekt komercyjny, sponsorowany przez firmę farmaceutyczną, jest to obecnie eksperyment medyczny, akademicki i nie istnieje możliwość zwrotu kosztów związanych z dojazdem do ośrodka prowadzącego badanie oraz kosztów jednodniowego pobytu w Warszawie związanego z wykonywanymi procedurami. Zgłaszając się na wizytę w pracowni medycyny nuklearnej, należy mieć ze sobą dokumentację oraz dokument potwierdzający tożsamość.

9. CZEGO SIĘ OD MNIE WYMAGA I OCZEKUJE?

Uczestnicy badania muszą zgłaszać się na planowaną wizytę oraz przestrzegać zaleceń i instrukcji lekarza prowadzącego badanie. Udział w badaniu nie nakłada żadnych ograniczeń na aktywność fizyczną ani inne aspekty życia codziennego.

Bardzo ważne jest udzielanie lekarzowi prowadzącemu badanie dokładnych i zgodnych z prawdą informacji na temat historii medycznej oraz wszelkich reakcji polekowych, które mogły kiedykolwiek u Pana/Pani wystąpić.

Udział w badaniu oznacza pewne ograniczenia, szczególnie w dniu wykonywania badania. Praktycznie nie ma ograniczeń w stosowaniu leków, rutynowo stosowanych przez Pana, dlatego należy informować lekarza o wszelkich lekach, które Pan przyjmuje lub planuje przyjmować. Odnosi się to także do leków dostępnych bez recepty, preparatów ziołowych i suplementów diety. Nie należy rozpoczynać żadnego innego leczenia (w tym preparatów tzw. medycyny naturalnej i suplementów diety) bez wcześniejszego skonsultowania się z lekarzem prowadzącym badanie.

Jeżeli wystąpią u Pana jakiekolwiek reakcje niepożądane lub problemy, należy skontaktować się z lekarzem. W takim przypadku lekarze postarają się przekazać Pana/Panią pod dalszą opiekę medyczną do ośrodka medycznego najbliższego zamieszkania.

10. JAKIE SĄ INNE MOŻLIWOŚCI DIAGNOZY

Jeżeli nie zechce Pan brać udziału w badaniu albo gdy zdecyduje się na udział, a następnie postanowi wycofać się z badania, lekarz omówi z Panem/Panią inne możliwe opcje postępowania. Lekarz omówi z Panem/Panią dostępne sposoby diagnostyki raka stercza, Szczegółowe informacje na ten temat zostaną omówione z lekarzem prowadzącym.

11. JAKIE NIEDOGODNOŚCI I ZAGROŻENIA MOGĄ WIĄZAĆ SIĘ Z UDZIAŁEM W PROJEKCIE

Udział w eksperymencie medycznym wymaga pewnego zaangażowania, tzn. Wykorzystania Pana jednego pełnego dnia, które zamierzony spędzić na dokonaniu wszystkich procedur wymaganych w/w projekcie naukowym. Niektóre konkretne zagrożenia związane z udziałem w obecnym badaniu opisane są poniżej:

11.1 Zagrożenia związane z pobieraniem krwi i podskórnymi wstrzyknięciami:

W miejscu wkłucia igły do pobrania krwi z żyły lub do wstrzyknięcia preparatu radioizotopowego może wystąpić ból, krwawienie, siniak i obrzęk. Pobieraniu krwi mogą towarzyszyć zawroty głowy lub omdlenia.

Próbki krwi będą pobierane z żyły w przedramieniu przy użyciu igły lub kaniuli - cienkiej plastikowej rurki zakończonej igłą, którą wprowadza się do żyły w przedramieniu.

11.3 Zagrożenia związane z napromieniowaniem pacjenta

Z uwagi na profil badania użycie diagnostyki radioizotopowej może wiązać się z wystąpienie działań niepożądanych związanych z napromieniowaniem narządów krytycznych takich jak nerki. Każdorazowo pacjent opuszczający Pracownię będzie miał wykonany pomiar dozymetryczny, opis powyżej.

Dodatkowo każdy pacjent udający się do domu transportem lotniczym lub wyjeżdżający poza granice kraju w kolejnym dniu po badaniu diagnostycznym z użyciem radioznacznika powinien poinformować lekarza prowadzącego badanie o takim fakcie z uwagi na kontrolę dozymetryczną prowadzoną przez Straż Graniczną.

11.4 Inne zagrożenia:

Z udziałem w badaniu mogą wiązać się jeszcze inne, nieprzewidziane jak dotąd zagrożenia. Zdrowie i bezpieczeństwo uczestników badania jest najważniejszym priorytetem dla lekarzy prowadzących badanie. Lekarz prowadzący badanie i jego zespół ponoszą odpowiedzialność za opiekę medyczną udzielaną Panu/Pani podczas badania i jeżeli w jakimkolwiek czasie uznają, że udział w badaniu może stanowić zagrożenie dla Pana/Pani zdrowia, wycofają Pana z badania.

12. JAKIE KORZYŚCI MOGĄ WIĄZAĆ SIĘ Z UDZIAŁEM W BADANIU?

Żywimy nadzieję, że proponowana diagnostyka radioizotopowa wg ustalonego schematu przyczyni się do poprawy skuteczność diagnostyki raka stercza i pomoże w rutynowym szerokim stosowaniu tej metody diagnostyki obrazowej. Informacje uzyskane dzięki temu badaniu mogą w przyszłości pomóc w specjalistycznej diagnostyce chorych na raka stercza

Tu kończy się Część 1.

Jeżeli zainteresowały Pana/Panią informacje przedstawione w Części 1 i bierze Pan pod uwagę udział w badaniu, prosimy przed podjęciem decyzji przeczytać dodatkowe informacje zawarte w **Części 2.**

Część 2

1. CO BĘDZIE, JEŚLI ZĘCHCĘ PRZERWAĆ UDZIAŁ W BADANIU?

Może Pan w każdej chwili zdecydować się na przerwanie udziału w proponowanym badaniu. Z uwagi na jednorazowy udział w badaniu opcja taka jest rozważana przed bezpośrednim badaniem, które jest prowadzone w ciągu jednego dnia roboczego. Ważne jest poinformowanie lekarza prowadzącego badanie o takim zamiarze lub rozważaniu takiej decyzji.

Jeżeli nie zdecyduje się Pan na uczestniczenie w badaniu lub postanowi przerwać udział, lekarz omówi z Panem/Panią właściwe opcje terapeutyczne w ramach państwowej opieki medycznej.

Udział w badaniu może zostać przerwany przez lekarza bez Pana zgody w następujących przypadkach:

- nieprzestrzeganie przez pacjenta zaleconego schematu postępowania określonego w protokole badania, wcześniej przedstawione i omówione z chorym;
- wystąpienie ciężkich działań niepożądanych SAE w trakcie trwania badania;
- lekarz uzna, że jest to dla pacjenta najkorzystniejsze rozwiązanie

2. CO BĘDZIE JAK POJAWI SIĘ PROBLEM?

Wykwalifikowany i posiadający ogromne doświadczenie w diagnostyce radioizotopowej personel prowadzący badanie w Pracowni Medycyny Nuklearnej, Uniwersyteckiego Szpitala Klinicznego (USK) w Olsztynie oraz w Centrum Diagnostyczno-Leczniczym Gammed w Warszawie, zapewni Państwu opiekę w razie problemu zdrowotnego w ramach powszechnego ubezpieczenia zdrowotnego.

3. SKARGI I ZAŻALENIA:

Jeżeli zaniepokoi Pana/Panią jakikolwiek aspekt badania, należy porozmawiać z osobami z zespołu badawczego, które postarają się jak najdokładniej odpowiedzieć na Pana/Pani pytania. Numery telefonów podane są na końcu tego dokumentu. Jeżeli nadal ma Pan/Pani wątpliwości lub obawy, można zasięgnąć porady u Rzecznika Praw Pacjenta pod numerem 0800 180590.

Jeżeli pragnie Pan/Pani skierować sprawę dalej, należy skontaktować się z rzecznikiem praw pacjenta NFZ,

4. UBEZPIECZENIE

Badanie jest eksperymentem medycznym, w którym ograniczona liczba uczestników rekrutowanych do tego projektu ogranicza zasięg badania. Badanie jest badaniem akademickim i nie jest badaniem komercyjnym – sponsorowanym. W ramach obowiązującego prawa nie jest wymagane na tym etapie obowiązkowe ubezpieczenie zespołu badawczego. Badanie będzie prowadzone zgodnie z wytycznymi Międzynarodowej Konferencji Harmonizacji (ICH) dotyczącymi dobrej praktyki klinicznej (GCP – Good Clinical Practice). Jeżeli jednak poniesie Pan/Pani szkodę na zdrowiu w wyniku przyjmowania badanej substancji lub jakiejkolwiek innej procedury zastosowanej zgodnie z protokołem, istnieje możliwość odszkodowania na podstawie posiadanej przez podmiot prowadzący badanie ogólnej polisy ubezpieczeniowej.

5. CZY MÓJ UDZIAŁ W BADANIU BĘDZIE TRAKTOWANY JAKO POUFNY?

Tak. Stosujemy się do wytycznych etyki i prawa i wszelkie dotyczące Pana informacje będą traktowane jako poufne. Jeżeli wyrazi Pan zgodę na udział w badaniu dane Pana mogą być przeglądane przez osoby prowadzące badanie w danej placówce. Pana dane osobowe mogą być ujawnione poza ośrodkiem tylko w ramach konsultacji lekarskich oraz na wniosek organów nadzorujących tego typu badania kliniczne czy eksperymenty medyczny oraz odpowiedniej Komisji Bioetycznej na jej wniosek.

Lekarz prowadzący badanie skontaktuje się z Pana lekarzem pierwszego kontaktu/lekarzem rodzinnym, aby go poinformować o Pana udziale w badaniu. Podpisując załączony formularz zgody, udziela Pan zgody lekarzowi prowadzącemu na skontaktowanie się z lekarzem pierwszego kontaktu/lekarzem rodzinnym.

Pana dokumentacja medyczna będzie przez cały czas traktowana jako ściśle poufna i taką samą poufnością będą objęte wszystkie dotyczące Pana informacje zgromadzone w ramach badania. Dokumentacja badania będzie udostępniona do wglądu tylko przedstawicielom organów nadzorujących tego typu badania w Polsce oraz w EU dodatkowo przedstawicielom organów regulujących obrót lekami w Polsce i Unii Europejskiej (Europejska Agencja ds. Leków oraz w USA (Amerykańska Agencja ds. Żywności i Leków) oraz wszelkich innych mających zastosowanie organów regulacyjnych w Europie.

Wszystkie strony obowiązuje przestrzeganie poufności. Jeżeli zgodzi się Pan na udział w badaniu i podpisze formularz zgody, udzieli Pan tym samym zgody wyżej wymienionym osobom na dostęp do Pana dokumentacji, nawet w przypadku, gdy wycofa się Pan z badania w późniejszym terminie.

Dokumentacja obejmuje dane dotyczące obecnego badania, jak również dane, które mogą być wykorzystane w przyszłych badaniach klinicznych prowadzonych w ramach tego samego programu badawczego. W odniesieniu do udostępnionych informacji zostanie zachowana całkowita poufność. Udział w badaniu jest poufny i Pana dane osobowe pozostaną anonimowe.

Wszystkie dotyczące Pana informacje zgromadzone w ramach badania, w tym dane osobowe oraz mogące Pana zidentyfikować próbki krwi i moczu, będą oznakowane wyłącznie przy użyciu numeru pacjenta, który zostanie Panu przydzielony na początku badania. Pana dane osobowe oraz próbki krwi i moczu zostaną w ten sposób zakodowane, a dostęp do klucza do kodu będzie odpowiednio zabezpieczony. Tylko lekarz prowadzący badanie będzie miał dostęp do tej informacji.

Dotyczące Pana dane będą przetwarzane elektronicznie w celu określenia wyników badania oraz przedłożenia danych regulacyjnym organom służby zdrowia. Dane te mogą też zostać wykorzystane do innych prac badawczych dla celów klinicznych związanych z obecnym badaniem. W tym celu Pana dane mogą być przekazywane do innych krajów. Podpisując formularz zgody, wyraża Pan zgodę na wysłanie takich informacji do krajów zarówno w UE, jak i poza Unią Europejską, w przypadku, gdy takie udostępnienie danych jest w opinii lekarza prowadzącego badanie konieczne dla celów właściwego prowadzenia badania lub innych celów związanych z badaniem. Odnosi się to do informacji przechowywanych, wykorzystywanych lub w jakikolwiek sposób ujawnianych.

Podmiot odpowiedzialny za badanie przestrzega wewnętrznych procedur dotyczących ochrony informacji osobowych, także w krajach, w których przepisy o ochronie danych osobowych są mniej rygorystyczne niż w Polsce. Gdy wyniki badania zostaną opublikowane, nie można będzie na ich podstawie Pana zidentyfikować. Wszystkie zapisane dane będą przechowywane przez co najmniej 15 lat od opublikowania końcowego raportu, zgodnie z wymogami organów ochrony zdrowia.

6. CO SIĘ STANIE Z POBRANYMI PRÓBKAMI PODCZAS BADANIA?

Wszystkie próbki i uzyskane na ich podstawie informacje będą przetwarzane bez użycia imienia i nazwiska, daty urodzenia ani innych danych, które mogłyby Pana bezpośrednio zidentyfikować. Pobrane od Pana próbki krwi i moczu będą bezpiecznie przechowywane przez aplikujący podmiot lub wyznaczonego współpracownika firmy, przez co najmniej 15 lat, po czym zostaną zniszczone.

Analizy próbek mogą być przeprowadzane w trakcie obecnego badania lub też w przyszłych badaniach zajmujących się badaniem wpływu substancji badanej na diagnostykę raka prostaty W takich przypadkach prace badawcze mogą wymagać stosowania innych testów niż te, które zaplanowano obecnie do badań z użyciem pobranych od Pana próbek. Przeprowadzenie takich analiz może potrwać kilka lat, ale być może doprowadzi do opracowania skuteczniejszych terapii w przyszłości. W takim przypadku poprosimy Pana ponownie o zgodę. Może Pan w każdej chwili zażądać zniszczenia pobranych próbek, ale wyniki analizy danych zebranych do tego czasu będą nadal wykorzystywane.

7. JAK ZOSTANĄ WYKORZYSTANE WYNIKI BADAŃ?

Po zakończeniu badania i zebraniu wszystkich danych zostanie sporządzony raport, a wyniki opublikowane w piśmie medycznym lub naukowym i/lub przedstawione na konferencjach naukowych oraz wykorzystane do dalszych badań. Wyniki będą opublikowane w formie anonimizowanej, aby nie można było zidentyfikować uczestników badania. Miejsce publikacji będzie znane dopiero w późniejszym terminie. Można będzie skontaktować się z lekarzem prowadzącym badanie, aby otrzymać kopię raportu z wyników.
Uczestnicy badania nie będą identyfikowani w żadnym raporcie ani publikacji.

8. KTO ZAAPROBOWAŁ TO BADANIE?

To badanie zostało zaaprobowane przez niezależną Komisję Etyczną Collegium Medicum, UWM w Olsztynie.

9. KONTAKT W PRZYPADKU DALSZYCH INFORMACJI?

Jeżeli macie Państwo jakiekolwiek pytania na temat badania lub leczenia, macie Państwo wątpliwości lub potrzebujecie dalszych informacji na jakikolwiek temat należy skontaktować się z lekarzem prowadzącym badania pod następującym numerami:

Dr hab. n. med. Jarosław B. Ćwikła Prof. UWM **telefon 602112599**
jbcwikla@interia.pl; lub jaroslawbcwikla@gmail.com
Dalsza informacja
Pracowania Medycyny Nuklearnej USK Olsztyn **telefon 895245648**
e-mail zmnolsztyn@gmail.com

Centrum Diagnostyczno-Lecznicze - Gammed **telefon 228223001**
www.gammed.pl, **komórka 507089942**
Gammed@gammed.pl **fax 22 8220315**

Opracował: Dr hab. n. med. Jarosław B. Ćwikła, Prof. UWM

FORMULARZ ŚWIADOMEJ ZGODY PACJENTA

„Diagnostyka przerzutów raka prostaty technikami SPECT i WB z wykorzystaniem ^{99m}Tc-PSMA-T4"

Nazwisko badacza: Dr hab. n. med. Jarosław B. Ćwikła Prof. UWM w Olsztynie

Proszę zaznaczyć poprzez wpisanie swoich inicjałów we wszystkie pola:

1) Potwierdzam, że przeczytałem i rozumiem broszurę informacyjną w wersji 1.0 z dn. 07.12.2018 dla powyższego projektu Miałem szansę przemyśleć te informacje i zadać pytania, na które udzielono mi satysfakcjonującej odpowiedzi.	
2) Rozumiem, że mój udział jest dobrowolny i mogę w każdej chwili zrezygnować bez podawania powodu. Nie będzie to miało wpływu na moją opiekę medyczną ani na moje prawa.	
3) Zgadzam się na udział w badaniu oraz na przewidziane badaniem procedury oraz wgląd w moją dotychczasową dokumentację medyczną dotyczącą mojej choroby, pod warunkiem, że moja anonimowość zostanie zachowana.	
4) Rozumiem, że anonimowa dokumentacja mojej diagnozy, mojego leczenia i wywiadu będą przekazane zespołowi badawczemu, ale wszystkie takie informacje będą anonimowe.	
5) Rozumiem, że część notatek medycznych o mnie, mogą zobaczyć odpowiedzialne jednostki, organy regulacyjne i zgadzam się na to.	
6) Zezwalam, żeby zanonimizowane informacje o mnie były trzymane w zabezpieczonych komputerowych systemach.	
7) Potwierdzam zgodę na wzięcie udziału w badaniu.	

_______________________ ____/__________/_____ _______________________

Nazwisko Pacjenta *(Drukowane)* **Data: DD/MM/YYYY** **Podpis**

_______________________ ____/__________/_____ _______________________

Nazwisko osoby odbierającej zgodę **Data: DD/MM/YYYY** **Podpis**

1 kopia dla pacjenta, 1 w dokumentacji medycznej
