# Supplementary material for: Initial Experience of Clinical Use of [99mTc]Tc-PSMA-T4 in Patients with Prostate Cancer. A Pilot Study
Source: Pharmaceuticals (Basel). 2021 Oct 29;14(11):1107. doi: 10.3390/ph14111107 (PMC8623387; doi:10.3390/ph14111107)
Supplement: Supplementary file 1 [file pharmaceuticals-14-01107-s001.zip › JBCwikla_PSMA-T4_Supplementary-2021_09_09.pdf]

Supplementary data

Materials and Methods

Exclusion criteria

The major exclusion criteria were as follows: no histopathological confirmation of prostate cancer. Infection with hepatitis B virus (including carriers) during screening, i.e. hepatitis B positive surface antigen (HBsAg) or positive hepatitis C (anti-HCV) antibody. Within 6 months before inclusion into the study: myocardial infarction or other cardiac events requiring hospitalization (unstable angina, etc.); acute congestive heart failure or severe arrhythmia (like ventricular arrhythmia, 2nd or higher degree atrio-ventricular (AV) heart block. Cerebrovascular accident, transient ischemic attack, acute stroke etc. Subjects with pulmonary embolism or deep vein thrombosis have been reported within the last 4 months.

Study drug

Briefly, for the synthesis polystyrene Wang resin with Glu(tBu)-urea-Lys-NH2 was used and the Fmoc-L-Trp(Boc) was attached to the free amine group of Lysine, using 1-[(1-(cyano-2-ethoxy-2-oxoethylideneaminooxy) dimethylaminemorpholino)]uronium hexafluorophosphate (COMU). Protected lysine was activated and attached to hydroxyl group of Wang – polystyrene resin. After washing the resin, the Fmoc protecting group was removed using piperidine solution. Protected glutamic acid was reacted with triphosgene in anhydrous conditions to form isocyanate. The dry resin with lysine was added to the solution of isocyanate and stirred to react. The reaction produced the urea moiety attached to the resin. The Alloc group protecting the amine group of lysine was removed using palladium complex catalyst. Fmoc-LThr(Boc) and Fmoc-4Amc were coupled using COMU in a standard reaction conditions used for amino acid coupling. Then protected HYNIC was coupled using COMU with DIPEA as a base in room temperature. After the reaction, the resin was washed with solvent and dried under vacuum. The crude product was detached from the resin using concentrated trifluoroacetic acid solution and after precipitation and washing with diethyl ether, it was purified using preparative HPLC. Finally, the purity of the PSMA-T4 was analyzed by HPLC and molecular structure was characterized by ^{1}H,^{13}C–NMR, and mass (LCMS-IT-TOF) spectroscopies.

Image Acquisition

A LEHRS, parallel-hole collimator, with a single photopeak window (140.5 keV $\pm$15%) was used and all patients. Whole Body tomographic (WB-SPECT/CT) acquisition was performed using a 360° orbit, 60 projections, 24s per projection, 128x128 matrix size, no zoom, using a step-and-shoot technique. The tomographic WB-

SPECT/CT covered head, neck, chest, abdomen pelvis including iliac joints. Raw WB-SPECT data were reconstructed into 3-dimensional volumes and corrected for attenuation, scatter correction and resolution recovery correction on a Xeleris 4DR workstation (GE Healthcare, Milwaukee, WI; USA) using ordered-subset expectation maximization (OSEM) iterative reconstruction methods (4 iterations/10 subsets) and filtered using a Butterworth filter (1.0 critical frequency, 10 power).

Image Analysis

All data were analyzed from WB-SPECT/CT views using an Xeleris Q.Volumetrix MI application, where patients' demographic data were assigned to each scan (weight, height, administered dose calculated from measured pre-injection activity, post-injection residual activity with times of measurement), camera sensitivity was measured using planar method for dedicated collimator-isotope set. Described above values gave possibility to calculate units of SUVmax according to SUVlbm formula described as: SUVlbm = (SPECT image Pixels uptake (Bqml)) * (LBM in kg) / (Actual_activity *1000) for patients: LBM in kg = 1.10 * (weight in kg) – 120 * [(weight in kg) / (height in cm)]^2. Images converted to SUVlbm are displayed in units of g/ml. For SUVmax (SUVlbm) assessment VOI sphere with 15mm diameter (Xeleris Q.Volumetrix MI InfoTip tool) positioned in deposit cursor point was used to pick-up results.
